# Cookies enriched with anethole and secoisolariciresinol diglucoside from flaxseed and fennel seeds improve hypercholesterolemia, lipid profile, and liver functions: A pilot study

**DOI:** 10.1002/fsn3.3433

**Published:** 2023-05-15

**Authors:** Sana Noreen, Tabussam Tufail, Tanazzam Tufail, Huma Bader Ul Ain, Hafiza Madiha Jaffar, Chinaza Godswill Awuchi

**Affiliations:** ^1^ University Institute of Diet and Nutritional Sciences The University of Lahore Lahore Pakistan; ^2^ Department of Medicine Faisalabad Medical University Faisalabad Pakistan; ^3^ School of Natural and Applied Sciences Kampala International University Kampala Uganda

**Keywords:** fennel seeds, flaxseed, functional cookies, hyperlipidemia

## Abstract

Over millennia, flaxseeds and fennel seeds have captured greater attention owing to the broad spectrum of bioactive compounds and their respective therapeutic potential. They are well‐known therapeutic plants, frequently used in home treatments for a variety of medical conditions. The novelty of this pilot study is to assess the beneficial health effects of secoisolariciresinol diglucoside (SDG) and anethole‐based enriched cookies among hyperlipidemic patients. The result of sensory evaluation revealed that cookies with anethole and SDG (500 + 500 mg/day) were significantly acceptable in terms of color, texture, taste, and overall acceptability same as that of control. This study was performed among 34 patients with hyperlipidemia in a university‐affiliated hospital, Lahore, Pakistan. In this study, patients received dietary supplementation with anethole and SDG (500 + 500 mg/day) administered in cookies for 8 weeks. Patients were assigned into two groups, intervention (receiving anethole + SDG‐enriched cookies; *n* = 16) and placebo (*n* = 18), for 8 weeks. Both groups maintained the same diet and lifestyle. Pre‐ and postintervention weight, lipid profile, and liver enzyme levels were measured. Analysis of covariance and paired sample *t*‐test were used for comparing the two groups. After 8 weeks, a significant mean weight loss was observed in the intervention group (4.26%) as compared to the placebo group (0.3%). A significant reduction of TC (177.02 ± 5.14 mg/dL; *p* = .024), TG (150.19 ± 7.94 mg/dL; *p* = .032), and LDL (87.38 ± 3.58 mg/dL; *p* = .001) were compared to the control group and HDL level (57.09 ± 3.90 mg/dL; *p* = .035) were increased in the intervention group as compared to the placebo. Meanwhile, it had a minor improvement in AST (30.97 ± 2.95 U/L; *p* = .01), ALT (33.05 ± 1.52 U/L; *p* = .025), and ALP (112.15 ± 4.03 U/L; *p* = .03) among the intervention group. Thus, based on the results from the study, it can be said that anethole + SDG‐enriched bakery products could be developed as a functional dietary option for hyperlipidemia in developing countries like Pakistan.

## Graphical abstract

Anethole and secoisolariciresinol diglucoside in fennel seeds and flaxseeds have nutraceutical properties on weight, lipid profile, and liver functions. Bakery products with added anethole + SDG could be developed as a functional dietary option for hyperlipidemia in developing countries.

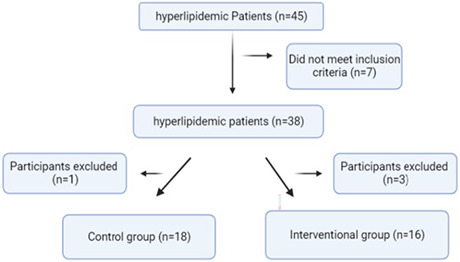

## 1 INTRODUCTION

The majority of naturally occurring antioxidative compounds include a phenolic component in their molecules. They can be found with catechines, tocopherols, and flavonoids. When combined with phenolic antioxidants, organic acids, carotenoids, protein hydrolysates, and tannins can serve as antioxidants or have a synergistic impact (AL‐Ansi et al., 2019). Fennel (*Foeniculum vulgare* Mill.), a fragrant plant, is commonly used as a food additive and for medicinal purpose (Shuja et al., 2022). Fennel seeds contain essential oils, including *trans*‐anethole (TA), estragole, and fenchone, making the entire plant vital in the pharmaceutical business (Destro et al., 2019). *Trans*‐anethole (1‐methoxy‐4‐(E)‐propenyl‐benzene) present in fennel seeds has a number of pharmacological properties, including antioxidant, anti‐inflammatory, antibacterial, antifungal, antiparasitic, antidiabetic, cardioprotective, hepatoprotective, antihyperlipidemic, anticancer, and estrogenic, which have been documented in earlier research (Samadi‐Noshahr et al., 2021; Shahbaz et al., 2023).

One of the first domesticated crops flaxseed (*Linum usitatissimum*) was used to make flour for bread as early as 1000 BC (Parikh et al., 2019). Due to its potential health advantages, notably for cardiovascular protection, flaxseeds are currently being more employed in human diets. The main lignan ingredient in flaxseeds is secoisolariciresinol diglucoside (SDG), making it the richest natural source of plant lignans (Kezimana et al., 2018). The phytoestrogens (flax seed lignans and soy isoflavones) are often found in the human diet. A number of studies employing dietary flaxseeds have been carried out to date, and the results showed that SDG lignan may reduce plasma cholesterol concentrations (Noreen, Rehman, et al., 2023; Nwozo et al., 2023; Zhuang et al., 2021). The purpose of this study was to evaluate the effectiveness of SDG + anethole‐enriched cookies for hyperlipidemic patients having high range of total cholesterol (>220 mg/dL), total triglyceride (>150 mg/dL), and low‐density lipoprotein (>130 mg/dL).

## 2 MATERIALS AND METHODS

### 2.1 Materials

Total cholesterol (TC), low‐density lipoprotein (LDL), very low‐density lipoprotein (VLDL), high‐density lipoprotein (HDL), aspartate aminotransferase (AST), alanine aminotransferase (ALT), and alkaline phosphatase (ALP) were estimated using standard kits (Merck Specialties Pvt. Ltd.). Plastic bags were obtained from Reynolds company. SDG and anethole were obtained from flaxseeds and fennel seeds bought from the local market of Lahore, respectively, and kept at the Food Science and Technology Lab (Lab. No. 101) of the University Institute of Diet and Nutritional Sciences (UIDNS), University of Lahore, Pakistan. Wheat flour, eggs, butter, baking soda, brown sugar, skimmed dry milk, and salt were obtained from local market of Lahore, Pakistan.

### 2.2 Settings

The study was conducted at the following settings: Food Science and Technology Lab (Lab. No. 101) of the UIDNS, University of Lahore, Dilawar Hussain Foundation, Diabetes Management Center, Lahore, Pakistan.

### 2.3 Preparation of extracts

Prior to sample preparation, all glassware was silanized in a 5% dimethyldichlorosilane in heptane solution, followed by deactivation in methanol. Flaxseeds were ground in a coffee grinder and either used for analysis or further defatted by Soxhlet extraction in n‐hexane for 8 h (ISO 659:1998) and oven‐dried to a consistent mass. Magnetic stirrer was used for mixing of milled flaxseeds in n‐hexane (1:5 w/v) at room temperature for 1 h to extract the oil, according to prior studies that utilized stirring with n‐hexane. After extraction, gas chromatography mass spectrophotometer (GC–MS) was used for quantification (Hanaa et al., 2017).

### 2.4 Preparation of cookies

Cookies were made using the straight dough technique by Riaz et al. (2020). In a Hobart Mixer (Model N‐50; Hobart Corp.), the composite flours were mixed with other baking ingredients for 3 min as mentioned by Table 1, then eggs and water were added, and the process was continued until foaming occurred (around 5 min at a speed setting of 2). Before adding the flour and baking powder, the components were blended to make a homogenous mixture. The cookies were baked for 10–15 min at 175°C in a preheated baking oven using a coated aluminum pan (Kenmore Co.). The daily cookie serving (1 cookie equaling 25 g) was placed in separate plastic bags after chilling for 60 min.

**TABLE 1:** Formulation of whole‐wheat and anethole + SDG composite doughs.

Parameter	Samples	
	*C*_0_	*C*_1_
Whole‐wheat flour (g)	400	360
Anethole + SDG (g)	0	40
Salt (g)	2	2
Brown sugar (g)	180	180
Butter (g)	300	300
Egg (1 piece)	55	55
Baking powder (g)	5	2
Skimmed dry milk (g)	58	58
Total weight (g)	1000	1000

### 2.5 Physical analysis of cookies

The width, thickness, and spread factor for cookies were determined every fortnight up to 45 days according to the method described by American Association of Cereal Chemists. Approved Methods Committee (2000).

### 2.6 Sensory evaluation

The sensory evaluation of the cookies was conducted using 10 trained panelists. Cookies were coded with two‐digit numbers and positioned randomly. Cookies were distributed at random and coded with two‐digit numbers. The sensory assessment sheet was given to the panelists who used a 9‐point hedonic scale (9 = *strongly liked*; 1 = *severely hated*) as described previously (Meilgaard et al., 1999) to grade the color, appearance, flavor, texture, general acceptability, and affordability in accordance with their preferences. The panelists rinsed their taste receptors with mineral water and expectorant cups during the sensory examination (Adeboye et al., 2016; Sidel & Stone, 1993; Amagwula et al., 2022a, 2022b).

### 2.7 Subjects

**FIGURE 1:**
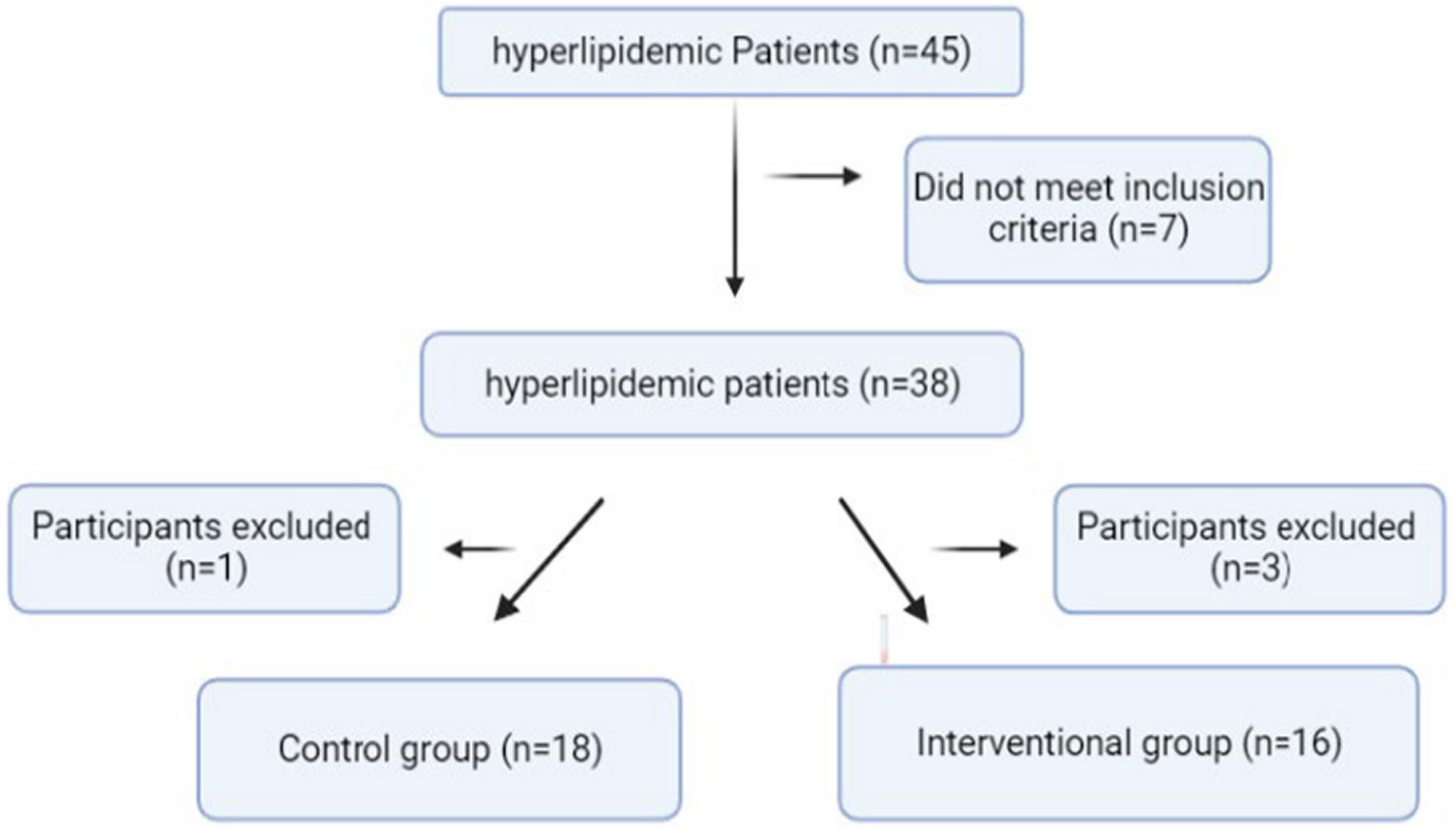
Consort form.

In this pilot trial, 45 patients signed up and seven people were eliminated because they did not meet the inclusion criteria (Figure 1). There were 38 patients in all who were randomized between the two groups. Of the 38 patients, three were removed from the intervention group due to travel, constipation, or the beginning of another medicine, while one patient left the placebo group to begin another medication. Sixteen participants in the therapeutic group and 18 participants in the placebo group eventually finished the trial and were evaluated after 8 months as mentioned in Figure 1. The patients took their existing medication as directed.

Participants who were diabetic, had serious illnesses or surgeries within the previous 3 months, or had secondary reasons for hyperlipidemia were not allowed to participate. Patients with gastrointestinal issues or documented sensitivities to fennel and flaxseed were also disqualified. All of the chosen participants were questioned in order to explain the experiment's purpose and to get their signed informed consent. The study comprised male and female participants aged 45–55 years. Patients having high range of total cholesterol (>220 mg/dL), total triglyceride (>150 mg/dL), and low‐density lipoprotein (>130 mg/dL) were included in this study. At the beginning of the trial, within the first week, and at the end of the experiment, after 8 weeks, blood was obtained to determine the baseline values for plasma lipids (Riaz et al., 2020). All subjects from the placebo and interventional groups consumed 25 g of cookies daily which contained the combination of SDG and anethole (500 + 500 mg) for the period of 8 weeks as shown in Table 2. Methods used were authorized by the Research Ethical Committee of the University of Lahore (IRB‐UOL‐FASH/826/2021).

**TABLE 2:** Treatment plan.

Groups	Placebo	Interventional
Dosage	Whole‐wheat cookie (25 g)	Whole‐wheat enriched cookie (25 g) anethole + SDG (1 g)
Frequency	Once a day	Once a day
Duration	8 weeks	8 weeks

### 2.8 Plasma lipids

Blood samples were collected for baseline and follow‐up testing in ethylenediaminetetraacetic acid (EDTA)‐treated tubes to assess the following hematological parameters: an enzymatic colorimetric method was used to quantify TC, triglyceride (TG), and HDL levels (Burstein et al., 1970). The LDL‐c concentration was measured using the formula of Friedewald et al. (1972). To calculate VLDL‐c, the following formula was used:

$$\text{VLDL}-c\;\left(\mathrm{mg}/\mathrm{dL}\right)=\mathrm{TG}/5$$

$$\mathrm{LDL}=\text{Total cholesterol}-\left(\mathrm{HDL}+\text{VLDL}\right)$$

### 2.9 Liver function assay

Serum AST, ALT, ALP were measured using an enzymatic colorimetric technique (Hafez et al., 2019).

### 2.10 Statistical analysis

The data were statistically processed by SPSS version 25. The mean ± SD values of numerical data like age, lipid profile, liver enzymes were presented. The quantitative variables between the two groups were compared using *t* test after fulfilling the parametric assumptions (Steel & Torrie, 2012).

## 3 RESULTS AND DISCUSSION

The study was carried out in two phases. The first phase included the physical and sensory evaluation of anethole + SDG‐enriched cookies, while the second phase assessed the effect of enriched cookies on lipid profile with human trials.

### 3.1 Physical parameters of cookies

There is no difference in width among control and fortified cookies as same standard fat was used in both groups (Table 3). Highest mean value for cookie width was observed in *C*_1_ (51.30 mm) as compared to *C*_0_ (51.12 mm). The mean values for cookies thickness revealed a significant difference between enriched and control cookies. The highest thickness value (7.14 mm) was noted in control cookies as compared to cookies enriched with anethole + SDG. Enriched cookies got minimum thickness (6.69 mm). Anethole and SDG caused the cookies to dry more quickly than previously thought because of the greater content of linolenic acid, which resulted in a reduction in cookie thickness (Masih et al., 2014). The flour's particle size index, moistness, and the type of fat used are the three main components that determine the spread factor of cookies while baking (Man et al., 2021). Spread ratio, which in turn is impacted by the relative water availability of various components and the capacity of wheat or any other component to absorb moisture during dough production, also has an impact on cookie diameter and thickness (Zhang et al., 2022).

**TABLE 3:** Physical parameters of wheat flour and enriched cookies (anethole + SDG extract).

Parameters	Samples	
	*C*_0_	*C*_1_
Width (mm)	51.30 ± 0.83	51.12 ± 0.92
Thickness (mm)	7.14 ± 0.10	6.69 ± 0.44
Spread factor	7.18 ± 0.73	7.64 ± 0.48

*Note*: The values are means of 10 replicates ± SD.

### 3.2 Sensory attributes of wheat flour and enriched cookies (anethole + SDG extract)

As indicated in Table 4, the sensory analysis of the fortified cookies revealed significant (*p* < .05) differences between control and fortified cookies in terms of texture, mouth feel, flavor, color, and general acceptance. A different pattern, reported by Sukhcharn (2008), emerged from the sensory assessment findings of the study. These varying score patterns might result from the panelists' varying rates of preference and acceptable values and the caliber of the generated completed cookies. One of the essential aspects of product quality is texture, which is the sensory expression of a cookie's structural makeup and how it responds to an applied force (Sruthi et al., 2021). Texture analysis measures the characteristics that affect how a cookie feels in the mouth (initial bite). The texture of the cookies in the control treatment (*C*_0_), which received the highest texture score, and those that included anethole + SDG extract (*C*_1_) significantly differed (*p* < .05), similar result was found by Khouryieh and Aramouni (2012).

**TABLE 4:** Sensory attributes of wheat flour and enriched cookies (anethole + SDG extract).

Types of cookies		Color	Appearance	Texture	Mouthfeel	Taste	Acceptability
Control	*C*_0_	8.18 ± 0.21	8.69 ± 0.14	8.91 ± 0.05	8.93 ± 0.15	8.23 ± 0.12	8.86 ± 0.01
Enriched cookies	*C*_1_	8.32 ± 0.12	8.23 ± 0.15	8.85 ± 0.07	8.24 ± 0.13	8.96 ± 0.09	8.09 ± 0.0

*Note*: The values are means of 10 replicates ± SD.

Taste is the key determinant of any product's acceptance and has the greatest influence on the product's commercial success (Mahmud et al., 2020). The flavor of the cookies differed considerably between the control and the treatment groups, according to the evaluation of the cookies' quality for taste. The findings showed that cookies prepared with 100% flour had a caramel flavor, while those created with anethole and SDG extract had a sweet flavor and scent that the presence of anethole may have caused. This can result from free sugars caramelizing during baking (Lee et al., 2022). The judges accepted cookies prepared from control and treatment of the composite flours containing SDG and anethole.

The color is a crucial factor in determining if cookies have been cooked properly since it not only shows what kind of raw materials were used in their production, but also tells you how they were made (Marand et al., 2020). The average quality score for the cookies' color is shown in Table 2. The data clearly show that cookies made from *C*_0_ scored lower, whereas cookies made from *C*_1_ with SDG and anethole scored higher. When exposed to sensory examination, judges approved the color of the cookies made with SDG and anethole. The level of SDG and anethole supplementation in the wheat flour was raised, which may have contributed to the dark brown color of the cookies, but it was okay. This resulted in a darker shade of brown in the cookies, but it was still acceptable. The mean scores were trending downward as the cookies' color shifted from light brown to dark brown. A possible cause of the deeper hue is the Maillard reaction between decreasing sugars and protein (Jiamjariyatam et al., 2022). A significant factor in the organoleptic estimate is the overall acceptability, which has many ramifications. According to similar findings published by Rathi and Mogra (2012), flaxseed‐based cookies were satisfactory up to a 70% supplementation level based on sensory qualities.

### 3.3 Clinical trial

After meeting the inclusion and exclusion criteria, 16 participants in the intervention group and 18 participants in the placebo group completed the trial, and the results were evaluated at the end of the 8‐week period. The results of the analysis revealed that the two groups were similar in terms of age and other fundamental characteristics as shown in Table 5a. The participants in the intervention and control groups were, on average, 47 and 49 years old, respectively. The findings of the study's pre‐ and postanalysis showed that there was a substantial drop in weight among the intervention group compared to the control group, showing that SDG‐and‐anethole‐enriched cookies were key in helping hyperlipidemic individuals lose weight in 8 weeks. The current results are consistent with those of earlier investigations by Baranowski et al. (2012), who discovered that the ALA and SDG content of dietary flaxseed oil, which lowers levels of protein‐1 (MCP‐1), adipocyte hypertrophy, inflammatory marker concentrations, and monocyte chemoattractant, is responsible for the effect of flaxseed oil on body weight. Fennel seeds are also excellent for managing metabolic disorders and weight. Anethole from fennel seeds was found to lower weight in hyperlipidemic individuals, according to a recent research. The same outcomes were also shown by AbdElwahab et al. (2021), who claimed that fennel seeds assisted in weight loss in hyperlipidemic obese individuals. In contrast, a prior study found that giving flaxseed powder supplements to hyperlipidemic rats had no appreciable impact on their weight gain (AbdElwahab et al., 2021).

**TABLE 5:** (a) Anthropometric measurement and (b) liver enzymes of hyperlipidemic patients fed control and anethole + SDG‐enriched cookies at baseline and after 8 weeks.

Parameters	Groups	Pretest	Posttest	*p*
(a)				
Age (years)	Placebo	47.83 ± 2.43		–
	Interventional	49.05 ± 3.35		–
Height (cm)	Placebo	166.64 ± 8.47		–
	Interventional	172.09 ± 9.89		–
Weight (kg)	Placebo	82.58 ± 1.37	83.05 ± 0.87	.029
	Interventional	86.49 ± 2.07	82.23 ± 1.52	.017
BMI (kg/m^2^)	Placebo	29.74 ± 0.49	29.91 ± 0.32	.029
	Interventional	29.20 ± 0.70	27.76 ± 0.51	.017
(b)				
ALT (U/L)	Placebo	50.23 ± 1.95	51.01 ± 1.67	.014
	Interventional	44.58 ± 1.32	33.05 ± 1.52	.025
AST (U/L)	Placebo	39.90 ± 1.99	40.99 ± 1.65	.009
	Interventional	36.20 ± 2.03	30.97 ± 2.95	.010
ALP (U/L)	Placebo	135.30 ± 2.78	135.85 ± 2.05	.010
	Interventional	135.78 ± 3.10	112.15 ± 4.03	.037

*Note*: The values are means of 18 replicates ± SD in placebo group and 16 replicates ± SD in interventional group. Significant value ≤.05.

However, liver enzymes significantly decreased in intervention group, AST (30.97 ± 2.95 U/L; *p* = .01), ALT (33.05 ± 1.52 U/L; *p* = .025), and ALP (112.15 ± 4.03 U/L; *p* = .03), while there was no improvement in placebo group as shown in Table 5b. Same results were also found by Elghazaly et al. (2019), who found improvement in liver enzymes with fennel seed consumption. The results of the current study, which involved treating hyperlipidemic patients with anethole + SDG‐enriched cookies, showed a significant improvement in liver function. These findings were consistent with reports by Yilmaz and Altuntaş (2020), who reported that hepatoprotective effects could be found in herbal remedies and the essential oils of fennel and flaxseed. In the current investigation, it was shown that individuals receiving conventional treatment saw no improvement in their liver enzymes; rather, there is marginal degradation in liver enzymes, but they are negligible (Yilmaz & Altuntaş, 2020). Those who are taking fortified cookies with anethole and SDG showed improvement in liver enzymes. Similar study by Elghazaly et al. (2019) showed that herbal treatment improved liver enzymes among hyperlipidemic rats.

### 3.4 Plasma lipid

The study's findings demonstrated that all patients who took part in the study had changes in their plasma levels of triglycerides, LDL and HDL cholesterol, and total cholesterol between the study's baseline and after 8 weeks of dietary therapy. The interventional group considerably reduced plasma total cholesterol (177.02 ± 5.14 mg/dL; *p* = .024), TG (150.19 ± 7.94 mg/dL; *p* = .032), and LDL (87.38 ± 3.58 mg/dL; *p* = .001) than the control group, as indicated in Table 6. As compared to the control group, significant improvement in HDL (57.09 ± 3.90 mg/dL; *p* = .035) was observed in interventional group. The results of this study are consistent with the findings from the previous study by Naik et al. (2018). There was no evidence of a beneficial impact of flaxseeds on lipid profiles in the study by Wu et al. (2010) on the relationship between lifestyle choices and flaxseed supplementation and metabolic syndrome. Due to higher exogenous cholesterol absorption and subsequent buildup, hyperlipidemic rats' plasma and hepatic cholesterol levels rose in addition to decreased cholesterol catabolism, as demonstrated by a reduction in bile acid formation and turnover of bile acids (Barakat & Mahmoud, 2011). In this current investigation, consumption of cookies enriched with anethole + SDG restored the lipid profile to normal ranges. Studies have suggested similar effects (Noreen, Rehman, et al., 2023; Noreen, Tufail, et al., 2023). An earlier which study found that rats on a high‐cholesterol diet with flaxseed oil supplementation had their blood lipid profiles improved was used to support this conclusion (Adolphe et al., 2010; Farook et al., 1991). They explained that ALA, which encourages cholesterol release into bile, depletes the intrahepatic pool of cholesterol and, as a result, increases cholesterol synthesis and metabolism. Furthermore, an ALA‐rich diet helps in reduction of hepatic lipid deposition both by stimulating β oxidation and by inhibiting fatty acid synthesis (Hussein et al., 2014). An earlier study by Zhang et al. (2022) also supported this study analyses as it showed significant reduction in TC (22%) and LDL‐c (24%) among hypercholesterolemic patients after 8 weeks of consuming 600 mg/day SDG derived from flaxseeds (Torkan et al., 2015). The same outcomes were obtained in a second research in which volunteers received a single 500 mg dosage of *trans*‐anethole for 7 weeks (12 mg/kg body weight) in order to manage hyperlipidemia in patients (Mehra et al., 2021). Therefore, the combination of anethole + SDG is proposed as a new approach for the therapeutic therapy of patients with hyperlipidemia.

**TABLE 6:** Effect of anethole + SDG‐enriched cookies among hyperlipidemic patients.

Parameters	Groups	Pretest	Posttest	*p*
TC (mg/dL)	Placebo	258.46 ± 2.69	206.10 ± 2.31	.055
	Interventional	263.27 ± 4.24	177.02 ± 5.14	.024
TG (mg/dL)	Placebo	253.82 ± 4.32	122.53 ± 3.94	.024
	Interventional	247.67 ± 3.98	150.19 ± 7.94	.032
LDL (mg/dL)	Placebo	176.18 ± 4.93	102.08 ± 2.73	.047
	Interventional	188.13 ± 4.76	87.38 ± 3.58	.001
HDL (mg/dL)	Placebo	39.00 ± 2.61	48.09 ± 2.51	.022
	Interventional	43.20 ± 3.32	57.09 ± 3.90	.035

*Note*: The values are means of 18 replicates ± SD in placebo group and 16 replicates ± SD in interventional group. Significant value ≤.05.

## 4 CONCLUSION

Anethole and SDG, active compounds of fennel seeds and flaxseeds, are extremely beneficial for positive health and help sustaining essential physiological functioning of the human body, and same has been validated by this study effort. Our study endorsed this fact by examining the lipid profiles of hyperlipidemic patients over the course of 8 weeks while they were fed anethole and SDG extract‐based cookies, which were successful in reducing TC, TG, and LDL concentrations in the interventional group. Due to this assurance, the ready‐to‐serve anethole + SDG‐enriched cookies, which are better referred to as “functional cookies,” may be made available to satisfy customer demand for a nutritious snack. In future, more studies can be performed on anethole + SDG based products for human welfare.

## AUTHOR CONTRIBUTIONS

**Sana Noreen:** Conceptualization (equal); data curation (equal); formal analysis (equal); investigation (equal); methodology (equal); project administration (equal); resources (equal); software (equal); visualization (equal); writing – original draft (equal). **Tabussam Tufail:** Conceptualization (equal); formal analysis (equal); project administration (equal); supervision (equal); visualization (equal); writing – original draft (equal); writing – review and editing (equal). **Tanazzam Tufail:** Conceptualization (equal); formal analysis (equal); investigation (equal); methodology (equal); project administration (equal); validation (equal); visualization (equal); writing – original draft (equal). **Huma Bader Ul Ain:** Conceptualization (equal); data curation (equal); investigation (equal); methodology (equal); project administration (equal); resources (equal); software (equal); supervision (equal); writing – original draft (equal); writing – review and editing (equal). **Hafiza Madiha Jaffar:** Data curation (equal); formal analysis (equal); project administration (equal); software (equal); supervision (equal); validation (equal); writing – original draft (equal); writing – review and editing (equal). **Chinaza Godswill Awuchi:** Data curation (equal); formal analysis (equal); methodology (equal); project administration (equal); resources (equal); supervision (equal); validation (equal); visualization (equal); writing – review and editing (equal).

## CONFLICT OF INTEREST STATEMENT

The authors declare no conflict of interest.

## ETHICAL APPROVAL

The methods used were authorized by the Research Ethical Committee of the University of Lahore (IRB‐UOL‐FASH/826/2021).

## Data Availability

The data used for this study are available on request through the corresponding author, though all the relevant data have been provided here.
